# Sleep complaints in adolescent depression: one year naturalistic follow-up study

**DOI:** 10.1186/s12888-014-0283-y

**Published:** 2014-10-08

**Authors:** Anna S Urrila, Linnea Karlsson, Olli Kiviruusu, Maiju Pankakoski, Mirjami Pelkonen, Thea Strandholm, Mauri Marttunen, the Adolescent Depression Study group

**Affiliations:** Department of Mental Health and Substance Abuse Services, National Institute for Health and Welfare, P.O. Box 30, , 00271 Helsinki, Finland; Department of Adolescent Psychiatry, Helsinki University Central Hospital, P.O. Box 83, , 00029, HUS Helsinki, Finland; Department of Child Psychiatry, Turku University Hospital, Kiinamyllynkatu 4-8, 20520 Turku, Finland; Department of Psychiatry, University of Helsinki, Institute of Clinical Medicine, Helsingin yliopisto, P.O. Box 20, , 00014 Helsinki, Finland

**Keywords:** Adolescent, Depression, Follow-up, Hypersomnia, Insomnia, Sleep

## Abstract

**Background:**

Sleep complaints are highly prevalent in adolescents suffering from major depressive disorder (MDD). The aims of this study were to describe the longitudinal course of sleep complaints, and to assess the association between sleep complaints and clinical outcome in a sample of adolescents with MDD during naturalistic follow-up.

**Methods:**

A sample of adolescent outpatients (n = 166; age 13–19 years, 17.5% boys) diagnosed with MDD was followed-up during one year in naturalistic settings. Sleep symptoms and psychiatric symptoms were assessed with interviews and self-report questionnaires.

**Results:**

All sleep complaints were less frequent at one-year follow-up compared to baseline. Baseline sleep complaints did not adversely affect clinical outcome at one-year follow-up: severity of the sleep complaints at baseline was associated with a steeper improvement of depressive and anxiety symptoms, suicidality/self-harm symptoms, and overall psychosocial functioning over time.

**Conclusions:**

Our results suggest that sleep disturbances at baseline do not necessarily lead to poorer clinical outcome during follow-up. Larger longitudinal studies combining both subjective and objective measures of sleep in depressed adolescents are needed to clarify the link between sleep and depression further.

## Background

Sleep complaints are highly prevalent in adolescents suffering from major depressive disorder (MDD) [1,2]. The presence of sleep disturbances has been linked with higher severity of depression, suicidal thoughts, comorbid anxiety disorders, and worse overall psychosocial functioning [1-3]. During recovery from depression sleep problems often persist: they rank among the most common residual symptoms in both adult [4-6] and adolescent patients [7-9].

In adults, subjectively and objectively measured sleep disturbances have been identified as risk factors for poor depression treatment outcome: they increase the risk of nonremission [10,11] and recurrence [12,13]. Further, in adults, residual sleep symptoms have been associated with various aspects of impaired quality of life, and residual nightmares have been associated with suicidal ideation [14].

Adolescents differ from adults and children in terms of both their natural sleep [15] and their typical characteristics of depression [16]. In depressed adolescents, the longitudinal course of sleep disturbances and their link with other features of depression has not been extensively studied. In a sleep polysomnography study of depressed children and adolescents, decreased sleep efficiency and delayed sleep onset predicted recurrences within one year [17]. A temporal relationship between sleep problems and completed suicide has also been observed in adolescents [18]. It has been suggested that sleep abnormalities would represent a persistent trait or vulnerability marker, rather than being a state dependent feature of depression in adolescents [19,20]. In a recent report, however, subjectively reported sleep disturbances were highly related to depressive state, and the persistence of sleep disturbance during the treatment phase was positively associated with depression at post-treatment follow-up [21]. It has been hypothesized that the concurrent treatment of depression and sleep disturbances in depressed adolescents would improve both sleep and depression outcomes, possibly even beyond the effects of traditional depression treatment [22].

Despite emerging new research, follow-up studies on sleep symptoms in depressed adolescents still remain scarce and knowledge on the extent to which sleep disturbances are associated with clinical outcome in adolescent depression remains limited. In particular, present data comes mostly from treatment studies, and naturalistic follow-up studies are lacking.

The aims of this study were: 1) to describe the longitudinal course of sleep complaints, and 2) to assess the association between sleep complaints and clinical outcome in a sample of adolescent outpatients with MDD during one-year naturalistic follow-up. We hypothesized 1) that all sleep complaints would be less frequent after the follow-up period compared to baseline,2) that sleep complaints would be associated with depressive state, poorer overall psychosocial functioning, anxiety symptoms, and suicidality/self-harm symptoms, and 3) that baseline sleep complaints would adversely affect clinical outcome.

## Methods

### Subjects

The sample comprised of n = 166 adolescent outpatients meeting the DSM-IV criteria for major depressive disorder (MDD) at baseline. The subjects were part of a larger study population (Adolescent Depression Study, ADS, n = 218) consisting of consecutive adolescent psychiatric outpatients with any DSM-IV depressive mood disorder diagnosis. As we were interested specifically in the relationship between unipolar MDD and sleep, subjects with any other depressive mood disorder diagnosis than unipolar MDD (e.g. bipolar disorder, dysthymia) were excluded from the analyses (n = 41). Further, subjects in full remission already in baseline (n = 8) and subjects with missing/inadequate data in baseline (n = 3) were excluded, leaving n = 166 adolescents in the analyses presented in this paper. The exclusion criteria of the ADS study included mental retardation, age under 13 or over 19 years, or insufficient knowledge of the Finnish language. The recruitment procedure has been described in detail in previous publications [23,24]. The subjects and their legal guardian, in case the adolescent was younger than 18 years, gave written informed consent to participate. The study protocol was approved by the ethics committees of Helsinki University Central Hospital and Peijas Medical Health Care District.

At baseline, the subjects were 13–19 years old (mean ± SD 16.5 ± 1.6 years), 17.5% of them were boys, and the level of depression was mild in 21%, moderate in 20%, severe in 46% and partially remitted in 13%. Most adolescents (70%) had at least one comorbid axis I diagnosis, of which anxiety disorders were the most common (57% of the adolescents). The sample characteristics as well as the prevalence of sleep complaints at baseline have been previously described in detail [1,23,24].

The study was naturalistic in nature and thus the adolescents received “treatment as usual” of clinically defined duration during the one-year follow-up period. Antidepressive medication was prescribed for n = 79 (55.6%) (serotonin selective reuptake inhibitors, SSRIs, for n = 76), anxiolytic medication for n = 44 (31.0%), nonbenzodiazepine sleep medication (z-drugs) for n = 25 (17.6%), and antipsychotic medication for n = 19 (13.4%). A minimum of 10 appointments of individual supportive psychotherapy was received by n = 112 (78.9%), and at least one family counselling session was received by n = 72 (50.7%). The majority of the adolescents (85.2%) received only outpatient treatment, but n = 15 (10.6%) were hospitalized once to an adolescent psychiatric ward and 6 adolescents had more than one period (2–4 periods) of hospital treatment.

The adolescents were re-evaluated approximately one year later (time between baseline and one-year follow-up 386 ± 35 days; mean ± SD). N = 142 adolescents (85.5% of the baseline sample; of which n = 24, 16.9% boys) completed the follow-up interview. The adolescents who did not attend the 1-year interview, did not show any statistically significant differences in clinical or sociodemographic characteristics at baseline as compared to the adolescents (n = 142) who completed the 1-year interview. Sleep data for more than one item was missing for n = 8 adolescents at the follow-up interview. At one-year follow-up, n = 49 (29.5%) of the adolescents had remitted from their baseline depressive episode with no recurrence, while n = 14 (8.4%) of the adolescents experienced a recurrent depressive episode and n = 79 (47.6%) had persistent depression. At one-year follow-up, two adolescents were diagnosed as suffering from bipolar type II disorder depressive episode, three from dysthymic disorder, and four from depressive disorder NOS.

### Assessment of psychiatric symptoms

During baseline and one-year follow-up interviews, present and lifetime episodes of DSM-IV **axis I disorders** were assessed with Schedule for Affective Disorders and Schizophrenia for School-Age Children—Present and Lifetime version (K-SADS-PL) [25]. *Remission* of MDD was defined according to the DSM-IV criteria as no significant signs or symptoms of MDD present during at least the past two months. *Recurrence* was defined as remission from the baseline MDD episode and onset of a new MDD episode [26,27]. The diagnoses (axis I-V) were confirmed in a diagnostic meeting where the original investigator and at least one senior clinician reached consensus on all measures of the interview, and inter-rater reliability was assessed using 13 randomly selected videotaped interviews (good inter-rater reliability for mood disorder diagnoses; described in detail previously [24].

Suicidality and self-harm symptoms were assessed as part of the K-SADS-PL interview, which includes a total of 5 items on suicidal thoughts, suicidal behaviour, and non-suicidal self-harm. Any type of self-harm symptom, when considered clinically significant in the interview, was taken into account in the analyses.

To assess overall psychosocial functioning, the global assessment of functioning scale (GAF; numeric range of 0–100) was used according to DSM-IV guidelines as part of the DSM-IV axial diagnostic procedure [28] during baseline and follow-up. GAF has been used also in studies among adolescents [29,30].

Anxiety symptoms were assessed with the Beck Anxiety Inventory (BAI) [31]. BAI is a 21-item self-report measure of anxiety symptoms with a maximum score of 63 points. It has been validated both in adults and adolescents [32,33].

The 21-item Beck Depression Inventory (BDI-21) is a standardized 21-item questionnaire to measure depression symptom severity [34]. It has been well studied also in adolescents [35,36]. The adolescents were asked to rate each of the symptoms on a 4-point scale ranging from 0 (Not at all) to 3 (Severely) according to the severity of the symptom (sum score range 0–63). In our analyses, the BDI-21 sleep item was excluded because we were looking at the relationship between sleep and depression symptom severity, and consequently the maximum score for BDI-21 in our analyses was 60 points. The Cronbach alpha value for BDI-21 at baseline in our sample was 0.912 when the sleep item was taken into account (21 items), and identical 0.912 when the sleep item was left out (20 items), showing that internal consistency was not compromised when leaving out the sleep item. The BDI-21 was performed during the baseline and follow-up interviews. In addition, the BDI-21 was used according to the clinician’s judgement several times between these two time points during the follow-up period. Measurements performed between 0–400 days from the baseline measurement were taken into account in the analyses. A total of 1300 BDI-21 measurements, giving an average of 7.8 (range 1–37) measurements per subject were performed during the follow-up period.

### Assessment of sleep complaints

The main source of information on sleep complaints was the K-SADS-PL attachment for assessment of affective disorders, which includes six items about the following sleep symptoms: initial insomnia, middle insomnia, terminal insomnia, sleep-wake rhythm disturbance, non-restorative sleep, and hypersomnia. The interviewer rates each symptom as non-existent (score: 1), sub-threshold (score: 2), or clinically significant (score: 3) according to standard criteria [1]. Insomnia was defined as suffering from clinically significant initial, middle, or terminal insomnia, or several of these insomnia symptom subtypes.

Additionally, we assessed nightmares as part of the 36-item General Health Questionnaire (GHQ-36) (answering scale: no – not more than usual – slightly more than usual – much more than usual) [37,38], since the presence of frequent nightmares has been previously linked with suicidality [39-41].

### Statistical analyses

Statistical analyses were performed with the IBM SPSS Statistics Version 21. To assess cross-sectional differences between subgroups, Chi-square tests and independent samples t-tests were used as appropriate. McNemar’s tests were used as non-parametric tests for categorical variables when comparing related samples (baseline vs. follow-up). A series of multiple linear regression analyses using the generalized estimating equations (GEE) method was conducted to assess whether sleep complaints at baseline were associated with clinical outcome measures (depression symptom severity, suicidality/self-harm symptoms, overall psychosocial functioning, anxiety symptoms) at follow-up. This method takes into account the within-subject correlation across repeated measurements [42]. For all analyses, a *p*-value of <0.05 was considered statistically significant.

## Results

### Prevalence of sleep complaints at follow-up

The sleep complaints at baseline have been described in detail in our previous paper [1]. At one-year follow-up, clinically significant sleep complaints (K-SADS-PL score: 3) were observed in 50.0% (n = 67) of the adolescents. The other half (n = 67) of the adolescents were free of clinically significant sleep complaints. The most frequent clinically significant sleep complaint (39.3% of the adolescents) at follow-up was non-restorative sleep. At follow-up, 13.4% (n = 18) of the adolescents suffered from a combination of several sleep disturbances (two or more of the following: insomnia, hypersomnia, sleep-wake rhythm disturbance). At the group level, all sleep complaints were less frequent at follow-up compared to baseline (McNemar’s tests *p* < 0.05) except for sleep-wake rhythm disturbance, in which the difference was close to but did not reach statistical significance (McNemar’s test *p* = 0.084).

### Sleep complaints and other features of depression at follow-up

**Figure 1:**
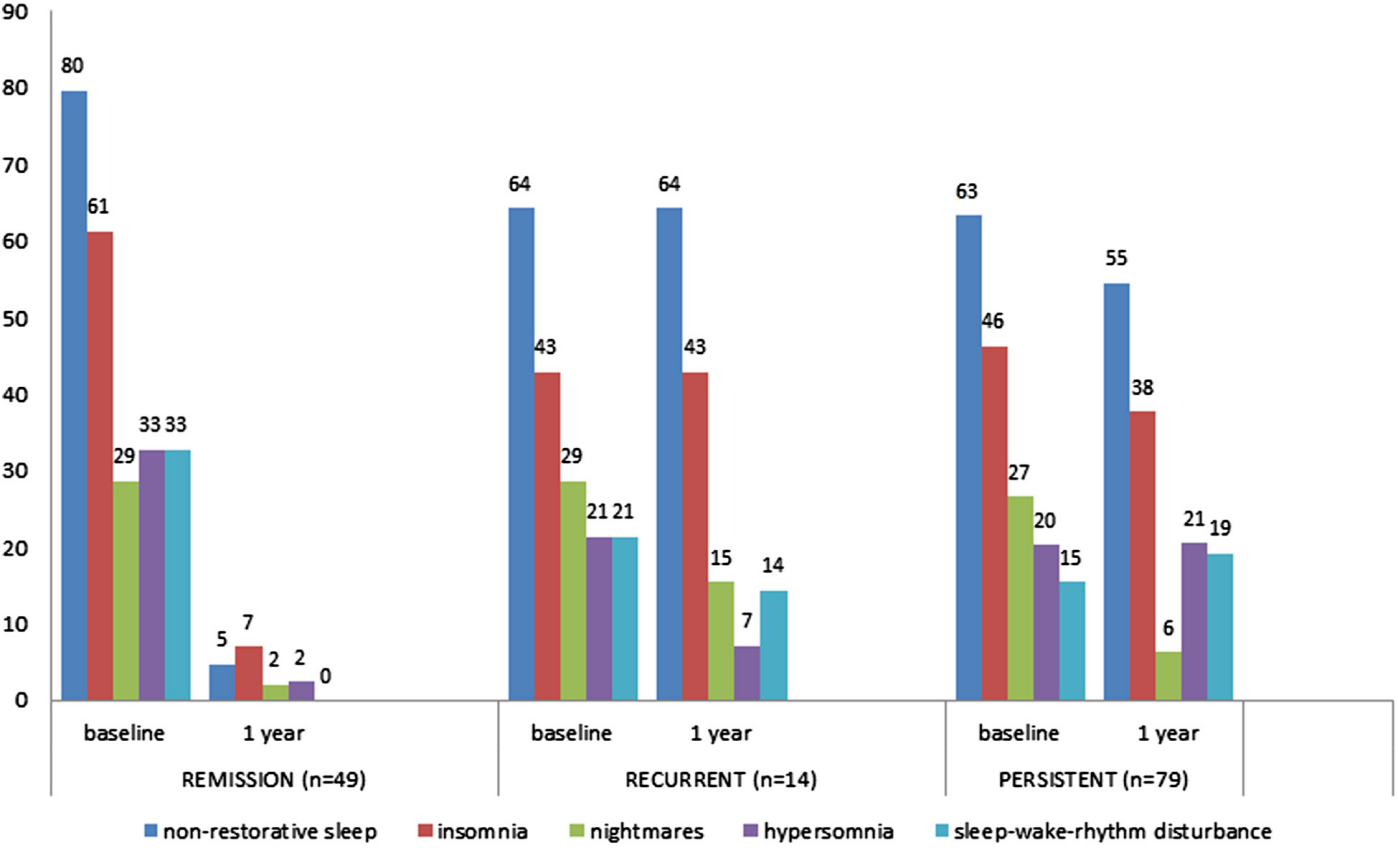
**The prevalence (% of the total sample; n = 142) of different sleep complaints among adolescents with MDD at baseline and at one-year follow-up.** The adolescents are grouped according to MDD status (remission, recurrent, persistent) at 1-year follow-up. Insomnia represents any significant complaints of insomnia (initial, middle or terminal). At one-year follow-up, sleep data for more than one item was missing for n = 8 adolescents.

At follow-up, sleep complaints were mainly observed in adolescents with persistent or recurrent depression (Figure 1). Sleep complaints were very rare in patients who had remitted from their baseline MDD episode: only three of them suffered from insomnia, two from non-restorative sleep, one from hypersomnia, and one from frequent nightmares. The recurrent and persistent subgroups did not differ from each other in terms of any sleep complaint (χ^2^ tests n.s.) except for middle insomnia, which was more common in adolescents with recurrent depression (χ^2^ = 8.37, *p* = 0.013).

At follow-up, adolescents suffering from any significant sleep complaint (n = 67) had higher scores in the BDI-21 (independent samples *t*-test t = 5.81; *p* <0.001) and BAI (t = 4.67; *p* < 0.001), and suffered more frequently from suicidality/self-harm symptoms (χ^2^ = 7.31, *p* = 0.006) compared to adolescents without sleep complaints (n = 67). No statistically significant difference was found in overall psychosocial functioning between these two subgroups (t = −8.37; *p* = 0.194). Suicidality/self-harm symptoms at follow-up were not connected with having much more nightmares than usual at follow-up or with having much more nightmares than usual at baseline (χ^2^ tests n.s.).

### The association of baseline sleep complaints with one-year clinical course

GEE models were used to investigate the association of baseline sleep complaints with depression severity, overall psychosocial functioning, anxiety symptoms, and suicidality/self-harm symptoms over time. For these analyses, we used the total sum of all six K-SADS sleep item scores at baseline (1–3 points per item, minimum 6 points, maximum 18 points; missing answers were substituted with the average points of all items) to form a continuous measure of sleep complaints (sleep complaint severity score). Age and gender were controlled in the analyses.

Main effects of sleep complaints were observed on the following outcomes: depression symptom severity (β = 1.15, standard error (SE) = 0.344, *p* = 0.001), anxiety symptoms (β = 1.03, SE = 0.373, *p* = 0.006), overall psychosocial functioning (β = −0.923, SE = 0.331, *p* = 0.005), and suicidality/self-harm symptoms (OR = 1.31, 95% Wald confidence interval (CI) [1.15-1.50], *p <* 0.001). Age was negatively associated with suicidality/self-harm symptoms (OR = 0.770, CI [0.640-0.927], *p* = 0.006), and female gender was associated with higher severity of depression (β = 2.95, SE = 1.29, *p* = 0.022).

An interaction between time and sleep complaint severity score was found in depression symptom severity (β = −1.36, SE = 0.364, *p* < 0.001), anxiety symptoms (β = −0.967, SE = 0.359, *p* = 0.007), overall psychosocial functioning (β = 1.50, SE = 0.457, *p =* 0.001), and suicidality/self-harm symptoms (OR = 0.681, CI [0.561-0.826], *p* < 0.001). (Table 1) These interactions indicate that the more the adolescent had sleep complaints at baseline, the quicker was the clinical improvement (steeper decline in depression/anxiety symptoms and suicidality/self-harm, as well as steeper improvement in overall psychosocial functioning).

**Table 1:** **Statistics on interactions between time and sleep complaint severity score (GEE model interactions)**

	**β** **/OR**	**SE/CI**	***p***
**BDI-21**			
time*sleep complaint severity score	−1.36	0.364	<.001*
**BAI**			
time*sleep complaint severity score	−0.967	0.359	.007*
**GAF**			
time*sleep complaint severity score	1.50	0.457	.001*
**suicidality/self-harm**			
time*sleep complaint severity score	0.681	[0.561-0.826]	<.001*

*denotes *p*-values <0.05, which were considered statistically significant.

All reported results concerning the effects of sleep complaints on clinical outcomes (main effects and interactions) remained significant also in additional analyses controlling for the baseline values of the outcomes.

The use of antidepressants, use of anxiolytics, use of antipsychotics, use of nonbenzodiazepine sleep medication (z-drugs), the amount of treatment appointments (cutoff ≥10 treatment appointments) during the 1-year follow-up period, or the amount of comorbid psychiatric disorders was not associated with the baseline sleep complaint severity score (independent samples t-tests n.s.). The adolescents who dropped out during follow-up (n = 24) did not differ from the adolescents who completed the 1-year interview (n = 142) in terms of their baseline sleep complaint severity score (independent samples *t*-test n.s.).

### Rate of depression symptom improvement

**Figure 2:**
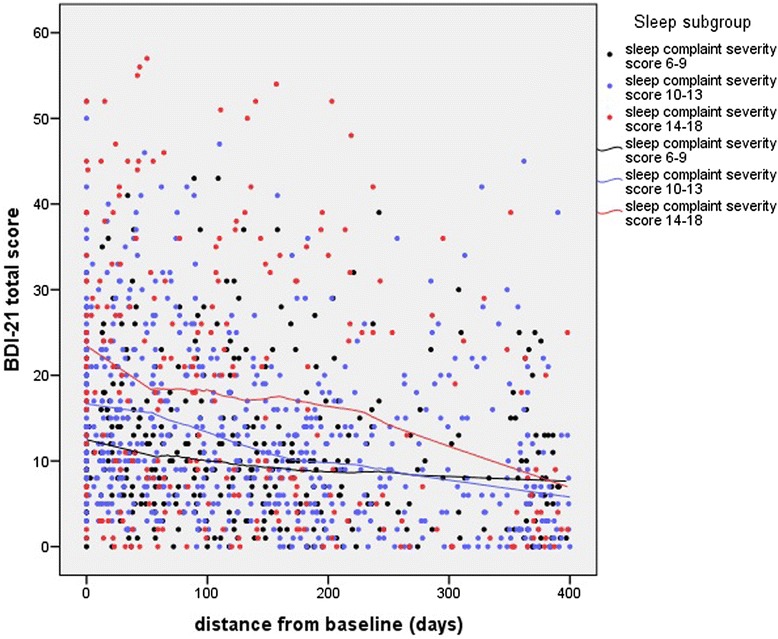
**BDI-21 total scores (sleep item excluded) during the follow-up period up to 400 days.** Black dots indicate subjects with a sleep complaint severity score of 6–9 (n = 52), blue dots indicate subjects with a sleep complaint severity score of 10–13 (n = 83), and red dots indicate the subjects with a sleep complaint severity score of 14–18 (n = 31) at baseline interview. Loess curves with respective colours are fitted for each of these sleep subgroups.

To assess the recovery process in more detail, the effects of baseline sleep complaint severity on the changes in the repeated BDI-21 measurement scores during the follow-up period were assessed with a GEE-model. Age and gender were controlled; female gender was associated with higher BDI-21 scores, while age showed no significant effect. Sleep complaint severity score at baseline showed a major effect on BDI-21 score. An interaction between time and sleep complaint severity score was observed (β = −0.003, SE = 0.0012, *p* = 0.028), indicating a significantly steeper decline in depression symptom severity over time in adolescents with higher sleep complaint severity score (Figure 2).

## Discussion

This is one of the first naturalistic clinical follow-up studies of depressed adolescents assessing the link between sleep complaints and clinical outcome. As hypothesized, all sleep complaints were less frequent after the one-year naturalistic follow-up period compared to baseline, and sleep complaints were associated with depressive state, poorer overall psychosocial functioning, anxiety symptoms, and suicidality/self-harm symptoms. Contrary to our expectations, however, baseline sleep complaints did not adversely affect clinical outcome at one-year follow-up.

Sleep complaints at follow-up were mainly observed in adolescents with persistent or recurrent depression and they were very rare in patients who had remitted from their baseline MDD episode. These findings support the tight link between sleep complaints and depressive state [21].

Residual sleep symptoms in adolescents who had remitted from depression were very rare. This result is in line compared to previous reports showing a ~3-12% prevalence of insomnia/sleep symptoms in adolescents remitted from depression [7,8,21]. Methodological differences, especially the length of the follow-up period (12 weeks of acute treatment vs. 1 year naturalistic clinical follow-up) may explain the dispersion of the results. A shorter follow-up time may not capture the full improvement of sleep symptoms over time. The comparison between different studies is also complicated by the heterogeneous ways of measuring and defining sleep disturbances. Our quite conservative method of assessing sleep disturbances, especially insomnia, may also have affected the results to some extent [1]. In our sample, sub-threshold residual sleep symptoms were approximately as common as having clinically significant sleep complaints among adolescents who had remitted from their baseline MDD episode (sub-threshold symptoms of insomnia were observed in n = 4 (9.30%), sub-threshold hypersomnia in n = 5 (11.6%), and sub-threshold sleep-wake rhythm disturbances in n = 2 (4.65%)).

Adolescents’ self-reported sleep estimates do not always correlate with objective measures of sleep [43-46]. It can be speculated that objective sleep abnormalities (like shortened REM sleep latency) previously reported in depressed children and adolescents in stable recovery [19] might have been present in our remitted subjects even in the absence of subjective sleep complaints. This possibility cannot be ruled out, but based on previous reports it seems, however, unlikely since even in the presence of subjective sleep complaints, sleep EEG has not been objectively disturbed in depressed children and adolescents [46]. The use of the K-SADS-PL to assess sleep disturbances is not the ideal approach to assess sleep disturbances, but it has been successfully used in previous studies [1,21,47], and it may provide valuable information on some aspects of sleep. Subjective measures of sleep are clinically useful and they convey the patient’s own experience of sleep.

Having many sleep complaints at baseline, although linked with a less favourable baseline status, was associated with a significantly quicker improvement of depression and anxiety symptoms and overall psychosocial functioning over time. This result was surprising because in previous studies in adults sleep disturbances have been identified as risk factors for poor depression treatment outcome. It is possible that the relationship between sleep and depression differs between adults and adolescents and is influenced by e.g. maturational factors and factors related to the stage of illness. It seems likely that clinical features other than disturbed sleep (including e.g. axis II comorbidity, MDD episode duration, excessive alcohol use) determined the one-year clinical outcome in our sample [23,48,49].

Differences in attrition rates, treatment efficacy or axis I comorbidities associated with sleep complaint severity could have influenced our findings. There were, however, no significant association between sleep complaint severity score and attrition rate, the amount of comorbid axis I disorders, or treatment received in our sample. The study was naturalistic in nature and treatment was not randomly assigned but planned according to clinical judgement, allowing also targeting treatment to sleep disturbances when necessary. Yet, no association between sleep complaint severity score and nonbenzodiazepine sleep medication (z-drugs) was observed. This may be explained by the fact that, apart from z-drugs (the only group of medication clearly targeted to treat sleep problems in our sample), many other groups of medications (such as benzodiazepines, sedative neuroleptics, mirtazapine, hydroxyzine etc.) may have been used to ameliorate sleep. Unfortunately we do not have data on the precise condition for which each medication was prescribed (e.g. a benzodiazepine may have prescribed to treat daytime anxiety or insomnia). At the time of data collection, no systematic sleep-related non-medication treatments (such as cognitive behavioural therapy for insomnia or chronotherapeutic treatments) were used in the Finnish adolescent psychiatric treatment units, but the adolescents may have been given advice on e.g. sleep hygiene measures during the treatment appointments.

Depressed patients with sleep disturbances may respond differently to both pharmacological and other treatment of depression compared to those without sleep problems [11,50-52]. The more detailed findings remain, however, mixed, and research evidence is lacking. Antidepressant drugs may ameliorate the sleep impairments in depression by e.g. inhibiting REM sleep [53], and especially patients with reduced REM sleep latency may respond favourably to antidepressants [54]. In a preliminary study, however, fluoxetine had a negative impact on sleep in depressed children and adolescents [55]. Depressed adolescents reporting insomnia have been observed to be less likely to respond to antidepressant treatment than those without insomnia [52]. In addition, depressed adolescents receiving medication for sleep have been found to respond less likely to depression treatment than those without sleep medication [51]. The low response rate of adolescents treated with sleep medication has been particularly associated with the use of trazodone (NB trazodone was not in use among our study sample), pointing towards drug-specific effects. [51] Further, the wide range of sleep disturbances (e.g. insomnia vs. hypersomnia) may interact with various depression treatments differently. Although the use of medication was not associated with sleep complaint severity score in our sample, we can not rule out the possibility that individuals with different types of sleep complaints may have responded differently to prescribed medications.

It has been suggested that joint treatment of disturbed sleep and depression may be more effective than treatment of depression alone [22]. The good one-year clinical outcome of our adolescents with baseline sleep disturbances may have been partly influenced by the naturalistic nature of the study: individual treatment choices were made based on the clinical picture, which allows taking also the precise characteristics of sleep symptoms into account. At the same time, though, in the naturalistic setting, the medication and other treatments received by the participants could not be tightly controlled, which complicates the interpretation of the data.

The relationship between sleep and mood is complex and bidirectional. One possibility is that at least for some of the adolescents the sleep disturbance may have been the driving force for depression, and the resolution of sleep problems may have also resulted in a reduction of depression symptoms.

Among the strengths of this study are the sample of clinically very well characterized subsequent outpatients, the naturalistic setting, the use of standardized evaluation instruments, and the homogenous age range. The study was not primarily designed to study sleep disturbances, but mood disorders. Thus, it did not include any objective characterization of sleep or any sleep-specific questionnaires, but we used the K-SADS-PL as the main source of information on sleep. This is the major limitation of the study. Further, we did not have explicit information on the sleep-related treatments the patients may have received during the follow-up period. Other limitations include the unequal gender distribution and the relatively small sample size.

## Conclusions

Our findings point toward a close link between sleep disturbances and depressive state in adolescents with MDD. Severity of baseline sleep complaints did not have an unfavorable effect on clinical outcome in a one-year naturalistic clinical follow-up, suggesting that sleep disturbances at baseline do not necessarily lead to poorer clinical outcome during follow-up. Larger longitudinal studies combining both subjective and objective (e.g. polysomnographic/actigraphic) measures of sleep in depressed adolescents should be conducted to clarify the link between sleep and depression in adolescents in further detail. Studies focusing on the management of the various sleep disturbances in depressed adolescents are also needed.
